# Anatomy of the Lisfranc joint complex: An illustrated review with surgical approach

**DOI:** 10.1002/ksa.70260

**Published:** 2026-01-22

**Authors:** Xiuqi Liu, Choon Chiet Hong, Josep Torrent, Javier Del Vecchio, Enric Alcolea‐Madera, Miki Dalmau‐Pastor

**Affiliations:** ^1^ Department of Orthopedic Surgery Affiliated Hospital of Zunyi Medical University Zunyi Guizhou China; ^2^ Human Anatomy and Embryology Unit, Department of Pathology and Experimental Therapeutics, School of Medicine and Health Sciences University of Barcelona Barcelona Spain; ^3^ Department of Orthopaedic Surgery National University Hospital, Singapore Yong Loo Lin School of Medicine, National University of Singapore Singapore Singapore; ^4^ Institut Rabat – Barcelona Foot and Ankle Clinic Barcelona Spain; ^5^ MIFAS by GRECMIP: Minimally Invasive Foot and Ankle Society Merignac France; ^6^ iMove Traumatology Tres Torres Sarrià‐Sant Gervasi Spain; ^7^ Cirurgia Ortopèdica i Traumatologia (COT) Hospital d'Olot i Comarcal de la Garrotxa Girona Spain; ^8^ Podiatry Unit, School of Medicine and Health Sciences University of Barcelona Barcelona Spain

**Keywords:** anatomy, Lisfranc, Lisfranc ligaments, midfoot, tarsometatarsal joint

## Abstract

This manuscript presents a detailed anatomical review of the Lisfranc joint complex, which includes the tarsometatarsal joints, the intertarsal joints and the proximal intermetatarsal joints. From the bony anatomy to the ligamentous structures reaching the base of the second metatarsal and the surgical anatomy relevant to fix the joint complex, this manuscript provides a comprehensive text for those treating Lisfranc injuries.

**Level of Evidence:**

N/A.

**Abbreviations**
C1: medial cuneiform
C2: intermediate cuneiform
C3: lateral cuneiform
EDB: extensor digitorum brevis
M2: second metatarsal
MCD: medial column dislocation
PE: proximal extension dislocation
TMT: tarsometatarsal

## INTRODUCTION

The Lisfranc joint complex is not a single joint but rather a complex anatomical–functional unit or a multifaceted polyarticular system of the foot, consisting of multiple skeletal elements, articular surfaces and ligaments with variable orientations, and defined by the interconnections between the metatarsal bases and the three cuneiforms as well as the cuboid. It primarily includes the tarsometatarsal (TMT) joints (the core component), the intertarsal joints and the proximal intermetatarsal joints, along with the associated ligamentous structures, which can be classified according to their location as dorsal, interosseous and plantar ligaments, followed by their fibre orientation as longitudinal, oblique and transverse [6, 9, 13, 25, 44]. According to this anatomic complexity, a Lisfranc injury is a broad term that refers to any fractures, dislocations or purely ligamentous sprains that affect any of these structures due to a low‐energy sports injury or a high‐energy injury, which can lead to partial or complete instability of the joint.

It should be noted that eponyms such as Lisfranc should not be used in anatomical terminology, as agreed and published in 1955 in the Nomina Anatomica Parisiensia [5]. Lisfranc described a surgical procedure for the amputation at the level of the TMT joint, and at some point, the joint itself was known as the ‘Lisfranc joint’ [19]. Finally, the terms ‘Lisfranc joint’ and ‘Lisfranc ligaments’ were used to refer to the joint and ligaments joining the second metatarsal (M2) and the medial cuneiform (C1), with its connecting Lisfranc ligaments (dorsal, plantar and interosseous). Its injury mainly involves disruption of one of these ligaments, leading to instability or dislocation of the first and second TMT joints, and is often associated with avulsion fractures (Fleck sign) or diastasis between the C1 and M2 base [12, 15, 38].

Based on the proximal extension of the injury involving the intercuneiform ligaments between the first and second cuneiforms (C1–C2), Lisfranc injuries can be further categorized into two distinct special subtypes: medial column dislocation (MCD) and proximal extension dislocation (PE). MCD involves injury to the Lisfranc ligaments and the intercuneiform ligaments between the C1 and C2, while the first TMT joint ligaments remain intact. On the other hand, PE represents more severe injuries, affecting more structures: the Lisfranc ligaments, the C1–C2 intercuneiform ligaments and also the ligaments of the first and second TMT joints (C1–M1 and C2–M2) [28]. Both subtypes are classified as longitudinal instability [14]. Due to its highly complex anatomy and critical role in load transmission, injury to the Lisfranc joint often results in foot biomechanical imbalance, functional impairment and even long‐term sequelae. In clinical practice, Lisfranc injuries without obvious fractures or dislocations, especially low‐energy injuries leading to isolated ligamentous injuries, are often overlooked or misdiagnosed (up to 20%) because of subtle symptoms and atypical imaging features. Delayed treatment can result in chronic midfoot instability, pain, arthritis (the commonest complication) and development of midfoot deformities [18, 21, 23, 30, 32]. The primary objective of this paper is to explore and highlight the anatomical characteristics of classical Lisfranc injuries and the associated variants with proximal extension, enhance understanding of these injury patterns, and support clinicians in accurate diagnosis and effective management to achieve optimal clinical outcomes.

## ANATOMY

### Skeletal elements

**Figure 1:**
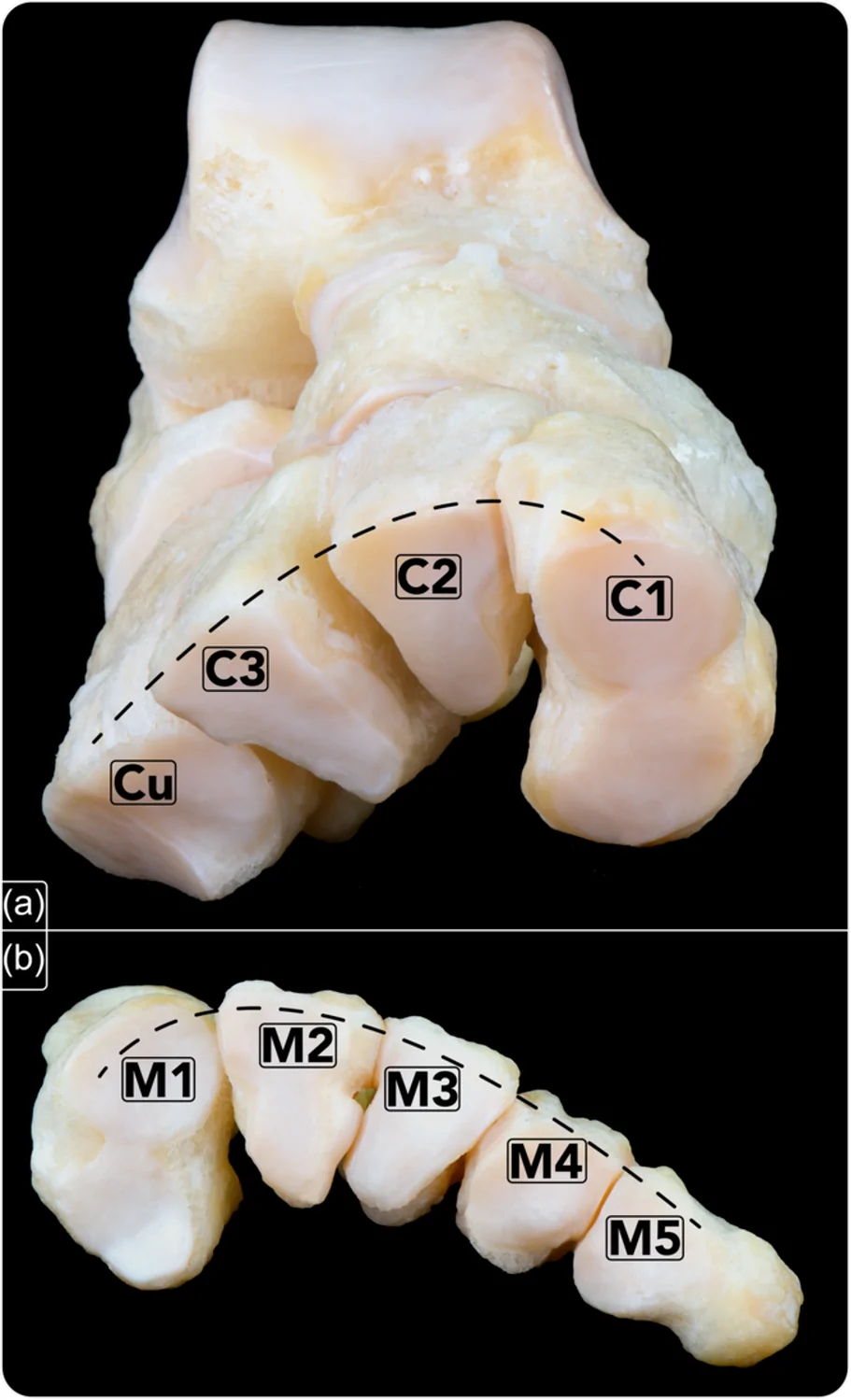
Arcuate disposition of the bony components of the TMT joint, with a dorsal convexity and a plantar concavity. (a) The anterior articular surfaces of the cuneiforms and cuboid bones. (b) The posterior articular surfaces of the five metatarsal bones. TMT, tarsometatarsal.

**Figure 2:**
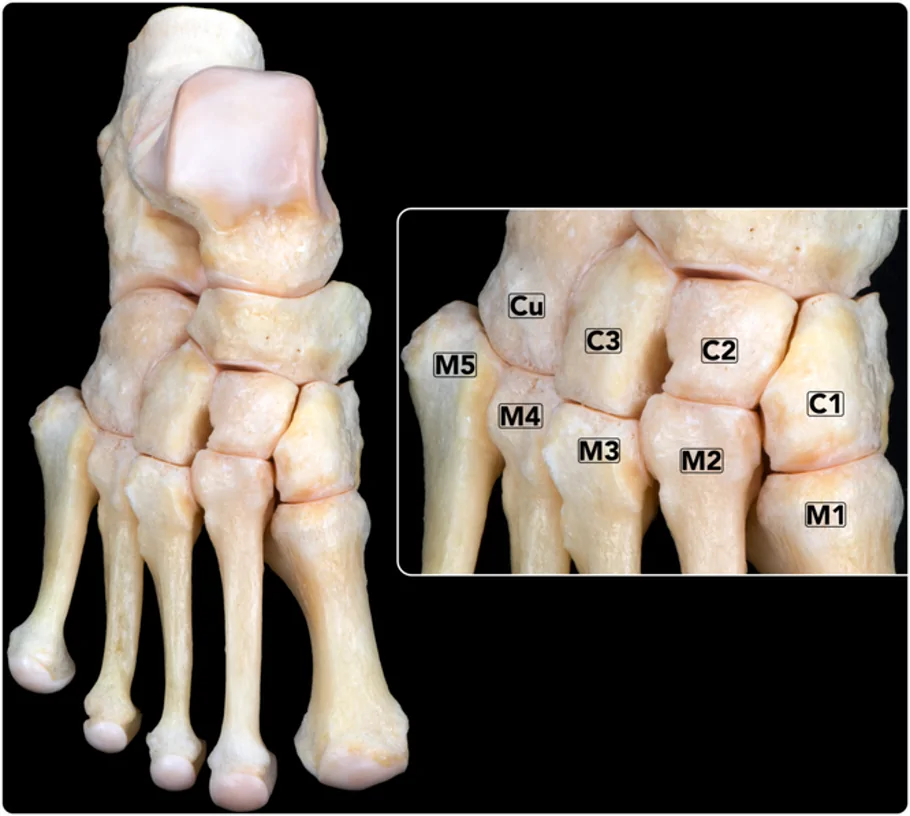
Dorsal view of the TMT joint. Notice the base of the second metatarsal, which is articulating with C1, C2, C3, M1 and M3. TMT, tarsometatarsal.

**Figure 3:**
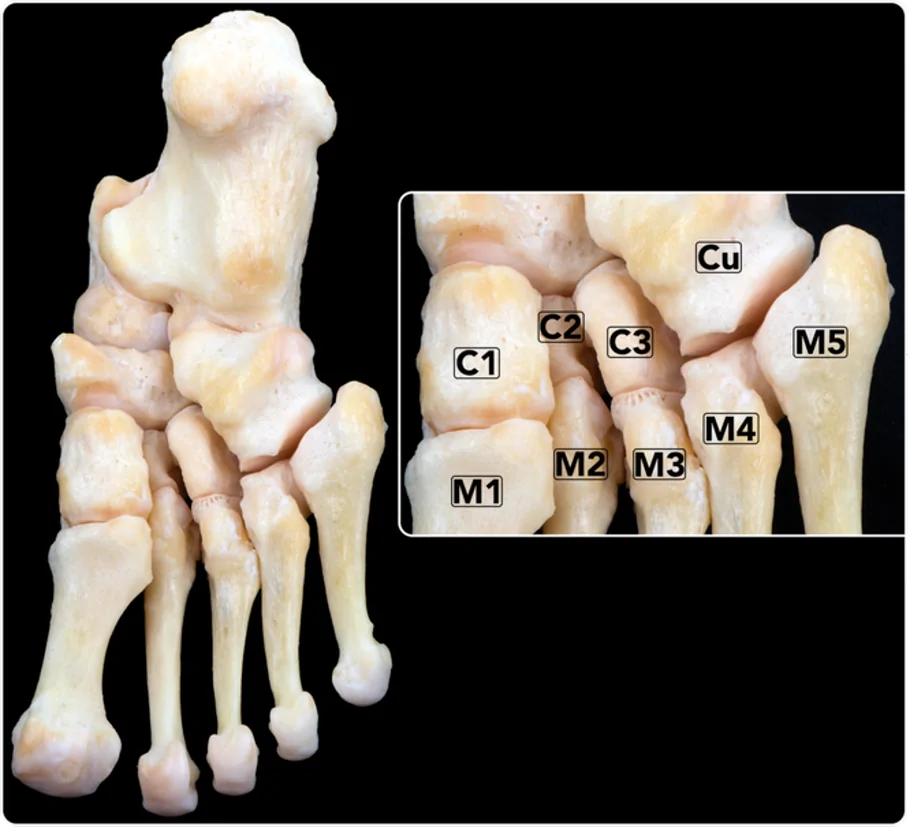
Plantar view of the TMT joint. The ‘cuneiform’ shape of the three cuneiform bones is clearly visible at C2, which has a small plantar surface. TMT, tarsometatarsal.

The TMT articular surfaces in the Lisfranc complex are arranged in a stepped and staggered manner, oriented outward and backwards from the base of the first metatarsal (M1), with an anteroposterior difference of approximately 20 mm [6]. This configuration creates an irregular, sinuous joint line that peaks at the third metatarsal (M3)—the third cuneiform (C3) articulation. The bases of the first to third metatarsals (M1–M3) are more or less trapezoidal or triangular in shape. A broad, flat dorsal surface forms the triangular base, with the medial and lateral surfaces converging on the plantar aspect to form the blunt apex [16]. This triangular morphology with a plantar vertex facilitates the formation of the ‘transverse arch’, which is dorsally convex and higher medially than laterally (Figure 1). Similarly, the C1, C2, C3 and the cuboid are also arranged to form a transverse arch. These arcuate arrangements provide substantial structural stability with little or no mobility, particularly as the base of the M2 wedges deeply between the C1 and C3 at the level of C2, serving as a key stabilizing element (Figure 2) [6, 9, 10, 11]. This region, which is located at the base of the M2, corresponds to the apex of the transverse arch and serves as a keystone in maintaining arch integrity. Notably, the C1 and C3 project anteriorly by approximately 8 and 4 mm relative to the base of M2, contributing to the mortise‐and‐tenon mechanism that underpins the structural stability of the joint complex [6, 16]. The C3 projects at least 2 mm anteriorly relative to the cuboid and wedges, to a lesser degree, between the bases of the M2 and M4, which creates a shallow mortise‐and‐tenon (Figure 3) [16].

The lateral surface of the C1 is approximately rectangular and contains two distinct articular facets. The facet for the base of the M2 is located along the anterior third of the superior border, extends to the anterior articular surface, and exhibits an oval shape. The facet for the C2 occupies the posterior two‐thirds of the superior border and the posterior edge, forming an inverted ‘L’‐shaped surface. The bases of the M1 and M2 typically do not articulate with each other. The medial articular facet of the M2 base, which corresponds to C1, is positioned dorsally, shaped like a segment of an arc, and continuous with the posterior articular surface [3, 6, 16]. Therefore, screw fixation across the C1–M2 joint for treating Lisfranc injuries carries a risk of iatrogenic damage to the articular cartilage.

Several anatomical factors may collectively contribute to the overall stability of the joint and influence its susceptibility to injury, including the medial depth of the mortise, the relative length of the second metatarsal, and the width of the mortise configuration [11, 26, 30, 44]. On one hand, there is a clear correlation between the medial depth of the mortise and the risk of Lisfranc injury. The greater the depth, the greater the joint stability, and the lower the risk of injury [26, 30, 44]. Specifically, each 1 mm increase in medial mortise depth reduces the likelihood of a Lisfranc injury by approximately 50%. In contrast, a shallower configuration increases the risk of joint instability because of the absence of a broader and stronger Lisfranc ligament to protect this joint complex [26]. On the other hand, the ratio of the second metatarsal length to the overall foot length demonstrates a significant association with the occurrence of Lisfranc injuries; specifically, for every 1% decrease in this ratio, the likelihood of a Lisfranc injury increases by a factor of 1.82. When the ratio falls below 29%, the joint may be particularly susceptible to this type of injury. A relatively shorter second metatarsal thus appears to be a potential predisposing factor for ligamentous or subtle Lisfranc joint injuries [11]. This parameter may serve as a valuable screening tool to identify individuals at increased risk of injury, thereby enhancing awareness and informing early decision‐making in occupational or athletic settings. In addition, the greater the width of this mortise configuration, the more robust the stability of the Lisfranc joint complex because of the broader ligaments, thereby reducing its possibility of injury [44]. However, the lateral depth of the mortise did not demonstrate a significant association with the risk of Lisfranc joint injury [26].

Assessing the distance between the bases of M1–M2, as well as the distance between C1 and the base of M2 on weight‐bearing CT and weight‐bearing radiographs, serves to evaluate Lisfranc injury and joint stability [21, 22, 29, 32]. Due to the position of the Lisfranc ligaments, the M1‐M2 distance does not accurately reflect the actual situation of Lisfranc ligament injuries. On the other hand, C1–M2 distance is a more precise and sensitive sign: an unstable injury is indicated if the distance is ≥2 mm [24, 29].

## LISFRANC LIGAMENTS AND INTERCUNEIFORM LIGAMENTS

**Figure 4:**
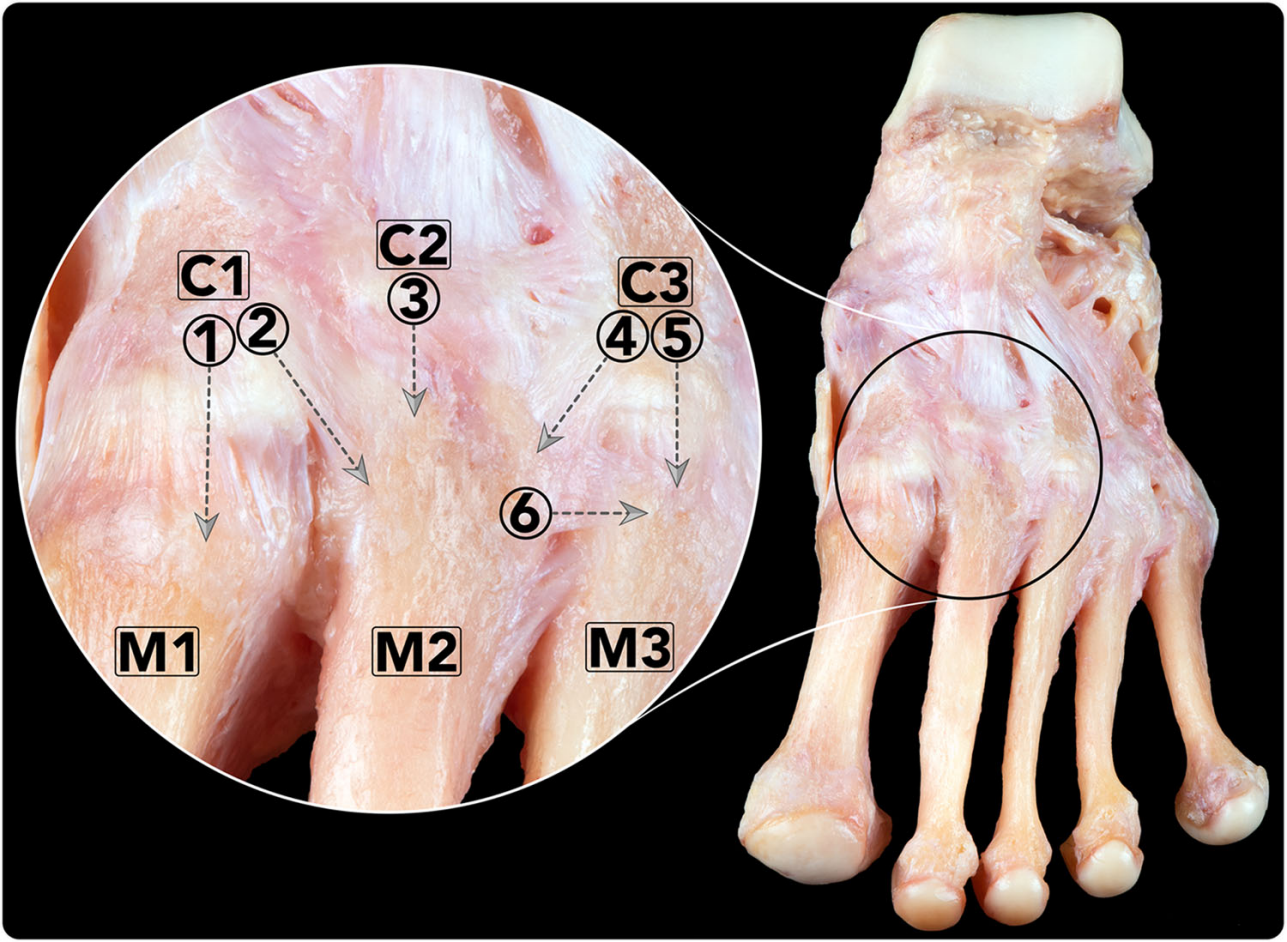
Dorsal view of an osteoarticular dissection demonstrating the ligaments stabilizing M2. (1) C1–M1 dorsal ligament. (2) Dorsal ‘*Lisfranc*’ ligament, joining C1–M2. (3) C2–M2 dorsal ligament. (4) C3–M2 dorsal ligament. (5) C3–M3 dorsal ligament. (6) M2–M3 dorsal ligament.

**Figure 5:**
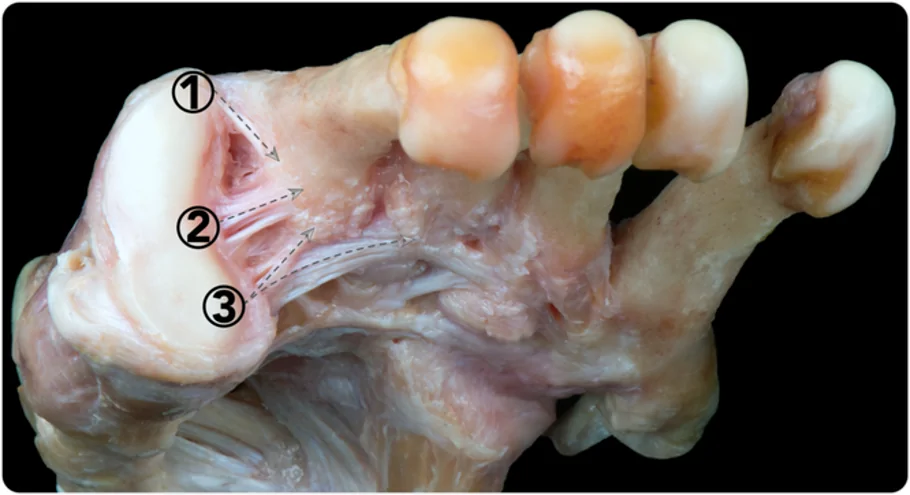
Anterior view of the ‘*Lisfranc*’ ligaments. M1 has been disarticulated to demonstrate the location of the C1–M2 ligaments. (1) Dorsal ‘*Lisfranc*’ ligament. (2) Interosseous ‘*Lisfranc*’ ligament. (3) Plantar ‘*Lisfranc*’ ligament, with a fascicle inserting on M2 and a fascicle inserting on M3.

The distribution of ligaments in the Lisfranc complex is not homogeneous: while the lateral metatarsal bases are united by dorsal and plantar ligaments, these ligaments do not exist between M1 and M2 (Figure 4). This is precisely why the ligaments that connect C1 to the base of M2, the dorsal (DLL), interosseous (ILL) and plantar Lisfranc (PLL) ligaments, are key stabilizing structures of the Lisfranc joint (Figure 5). At the interosseous level, the first and second metatarsal bases are only linked by weak fibrous tissue, making this region an inherent weak point particularly susceptible to injury [6, 16, 34].

The DLL, which originates from the lateral border of the dorsal surface of the C1 and inserts onto the proximal end of the medial border of the dorsal surface of the M2 [6, 12, 13, 40], is broad‐trapezoidal in shape [40] and has an average length, width and thickness of 10.8, 8.30 and 1.36 mm, respectively [7]. It is the thinnest of the Lisfranc ligaments, possesses the weakest tensile strength, and is thus the most susceptible to injury [7, 16, 17]. This explains why the resulting displacement occurs most commonly in a dorsal direction. The integrity of the DLL may reflect the status of the Lisfranc joint, and ultrasound screening can be a valuable tool for the early detection of subtle Lisfranc injuries [8, 20, 42].

The ILL arises from the lateral aspect of the C1, anterior to the intercuneiform ligament. It runs obliquely outward and slightly downward and attaches to the medial side of the base of the M2. It is band‐shaped and can vary in number from a single bundle to as many as four distinct bands. However, anatomical variations most frequently present as either single‐bundle or double‐bundle configurations [6, 12, 16, 25, 36, 41]. The main differences involve the attachment sites and the number of ligamentous bundles on the lateral surface of the C1. One variant features a single, thick ligamentous bundle that fans out, while the other comprises two bundles arranged anteriorly and posteriorly. Both variants share a single attachment site on the M2, with a mean attachment area of 135 mm^2^ [25]. It is the narrowest yet the thickest of the Lisfranc ligaments, with dimensions of 9.70 ± 2.11 [7], 8.02 ± 1.5 [1] and 5.9 ± 1.5 mm [36] in length; 1.66 ± 1.08 [7], 2.53 ± 0.61 [1] and 5.9 ± 3.2 mm [36] in width; and 13.74 ± 3.08 [7], 6.96 ± 1.01 [1] and 4.2 ± 1.3 mm [36] in thickness. Its thickness would provide the most significant surface area for fibroblast–fibronectin interactions, making it the strongest and primary stabilizing structure of the Lisfranc joint complex [7, 13, 34, 35]. On average, it measures 4.5 times the size of the DLL and is 2 times as large as the PLL [13], and also represents the largest and most robust of the interosseous TMT ligaments [16, 37]. It maintains the pivotal position of M2 within the midfoot arch [6].

The PLL is the main plantar stabilizing component of the Lisfranc joint complex. It extends obliquely outward and a little downward from the lateral anterior plantar surface of C1 to the proximal inferomedial portion of M2 and the plantar base of M3 [7, 25, 36, 39]. The PLL primarily exhibits two morphological patterns. The most common form is fan‐shaped, with its apex located on the C1, extending distally while widening to attach to the M2 and M3, and partially connected to the ILL. The less common variant showcased a split morphology, which is divided into the superficial band and the deep band, characterized by a single origin that bifurcates distally to separately insert onto M2 and M3 [4, 7]. The superficial band, which has a broad insertion at the base of the M3, forming a band shape, is both stronger and thicker, while the deep band is comparatively smaller and inserts into the base of the M2 [16, 39].

## INTERCUNEIFORM LIGAMENTS

**Figure 6:**
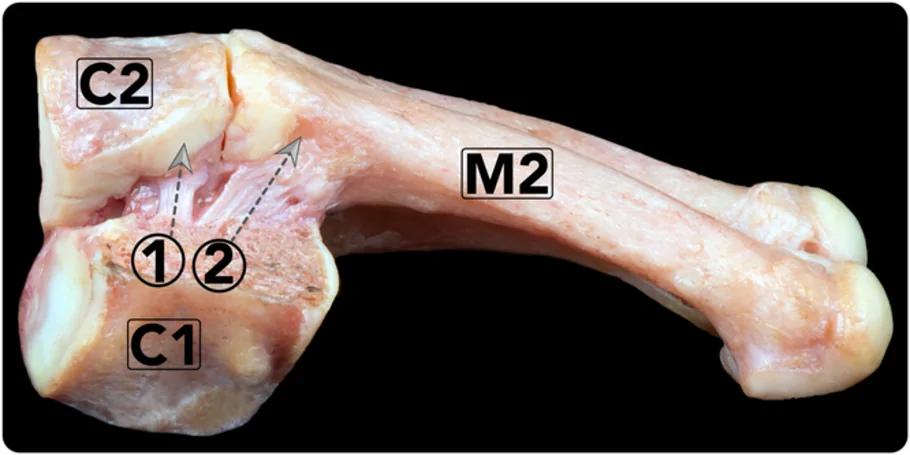
Medial view of an osteoarticular dissection of C1, C2 and M2. The dorsal half of C1 has been cut on the sagittal plane to show the location of (1). Interosseous C1–C2 ligament. (2) Interosseous ‘*Lisfranc*’ ligament.

The two intercuneiform joints connecting C1–C2–C3 are linked by the intercuneiform ligaments. These include two dorsal ligaments (C1–C2 and C2–C3), two interosseous ligaments (C1–C2 and C2–C3), and a single plantar ligament (C1–C2), with no plantar ligament connecting C2 and C3. Longitudinally unstable Lisfranc injuries can affect and disrupt the ligamentous structures between the C1 and C2. In this region, the dorsal ligament is relatively small and rectangular in shape, whereas the interosseous ligament, originating from the angulated junction of the ‘L’‐shaped articular surface of the lateral of the C1, is a robust, thick, and transversely oriented structure (Figure 6). The plantar ligament from C1 to C2 originates from the posterolateral aspect of the plantar surface of C1 and courses obliquely forward and laterally to attach to the plantar aspect of C2 [6, 16].

Lisfranc injuries can present as either transverse or longitudinal. Transverse instability results from the combined disruption of the ILL and the PLL. On abduction stress radiographs, both the C1–M2 and C2–M2 joints demonstrate instability. Longitudinal instability occurs due to disruption of the ILL and the interosseous ligament between C1 and C2. On adduction stress radiographs, instability of the C1–M2 joint is observed in one out of five specimens, while instability between the C1 and C2 is observed in four out of five specimens [14].

## BIOMECHANICS

An in vitro biomechanical study examining the mechanical behaviour of the ILL and DLL revealed that the stiffness of the DLL and ILL was 66.3 ± 18.3 and 189.7 ± 57.2 N/mm, respectively. The mean failure load was 150.7 ± 33.1 N for the DLL and 368.8 ± 126.8 N for the ILL, indicating that the ILL possesses significantly greater mechanical strength and structural resistance compared to the DLL [17]. In another biomechanical study of the Lisfranc ligament complex, the stiffness of the DLL, ILL and PLL was reported to be 40 ± 9, 90 ± 3 and 62 ± 3 N/mm, respectively. The average strength of the DLL, ILL and PLL was 170 ± 33, 449 ± 58 and 305 ± 38 N, showing that the ILL is the strongest and stiffest when force is applied in the direction of its fibres [33].

## SURGICAL ANATOMY

Surgical exposure requires careful consideration of the surrounding soft tissue envelope, which is thin and vulnerable to iatrogenic injury. The fracture configuration or injury pattern will determine the selection of surgical approaches.

Preoperative planning is essential in Lisfranc injuries to define the extent of instability. Key objectives include identifying unstable joints, determining the need for fixation or fusion, ensuring the adequate restoration of medial and lateral column lengths and addressing inter‐column ligamentous instability [27, 31].

In most cases, Lisfranc injuries involve the second TMT joint. The dorsomedial approach, made between the first and second metatarsals, provides access to the medial cuneiform and the first and second TMT joints [7].

When the third TMT joint is involved, or instability affects the fourth or fifth metatarsals, a double approach is recommended: we perform a dorsolateral approach aligned with the fourth metatarsal. It is essential to maintain a distance of at least 5 cm between these incisions to preserve the skin bridge [43].

### Surgical approaches and structures at risk

Dorsomedial approach: a longitudinal incision is made along the first intermetatarsal space, which can be extended proximally to include the talonavicular joint and distally to expose the entire first and second metatarsals. The main anatomical structures encountered are the extensor hallucis longus (EHL), the tibialis anterior tendon and the anterior tibial neurovascular bundle (dorsalis pedis artery and the deep peroneal nerve). The interval is developed between the EHL and the neurovascular bundle, which is carefully retracted laterally. This provides access to the first and second TMT joints. If exposure of the second metatarsal shaft is needed, dissection should be carried out lateral to the neurovascular bundle.

Dorsolateral approach: the incision is centred over the cuboid along the axis of the fourth metatarsal and can be extended proximally toward the tip of the fibula. The medial dorsal cutaneous nerve (branch of the superficial peroneal nerve) is often encountered in the dorsomedial approach. The key anatomical structures encountered are the extensor digitorum brevis (EDB), the peroneal tendons, and branches of the sural and superficial peroneal nerves, with special attention needed to protect the sural nerve. The EDB muscle belly is reflected dorsomedially, while the peroneal muscles are retracted plantarly. This dissection exposes the cuboid, and through the intermuscular plane between the EDB and the peroneal muscles, the lateral cuneiform and the bases of the third to fifth metatarsals can also be visualized. In Lisfranc injuries, achieving anatomic reduction of the joint complex is critical to reestablish stability [2]. Since these injuries almost always include an element of instability that may progress to deformity, the role of nonoperative treatment is very limited.

A general sequence for reconstruction begins with reestablishing the relationship between the navicular and the cuneiforms. Once this is achieved, the alignment of the M2 with the C2 and then the C1 is restored, as this step provides the cornerstone of reduction. The remaining TMT joints are then addressed sequentially, usually in the order of the first, third, fourth, and finally the fifth [21].

In summary, the Lisfranc joint complex is an intriguing architectural formation in the midfoot, allowing for a critical balance between stability and flexibility during gait and sporting activities. Its uniqueness lies in the arrangement of bony, articular, and ligamentous structures to ensure optimal alignment and load transfer from the hindfoot to the forefoot. This pictorial review highlights the critical structures and biomechanical features of the Lisfranc joint complex, which are imperative for accurate identification of Lisfranc injuries followed by early and appropriate intervention to allow optimal outcomes.

## AUTHOR CONTRIBUTIONS

Xiuqi Liu and Choon Chiet Hong wrote most of the manuscript, Josep Torrent and Javier Del Vecchio wrote the surgical approach part, Enric Alcolea‐Madera helped in the preparation of the specimens for the figures, Miki Dalmau‐Pastor did six revisions of the manuscript and prepared all the figures.

## CONFLICT OF INTEREST STATEMENT

The authors have nothing to report.

## ETHICS STATEMENT

The authors have nothing to report.

## Data Availability

Data Availability Statement is not available.
